# Structural Characterization and Bioactivity Evaluation of Selenium-Modified Dihydromyricetin from Vine Tea

**DOI:** 10.3390/foods14101735

**Published:** 2025-05-13

**Authors:** Kaixuan Cheng, Guangqian Hou, Shengqi Mei, Xingxing Gao, Chi Zhang, Longchen Shang, Shuai Chen

**Affiliations:** 1 Hubei Key Laboratory of Selenium Resource Research and Biological Application, Hubei Minzu University, Enshi 445000, China; 17335897915@163.com (K.C.); 202330386@hbmzu.edu.cn (G.H.); meishengqi0509@163.com (S.M.); zhtzu@163.com (C.Z.); 2 College of Biological and Food Engineering, Hubei Minzu University, Enshi 445000, China; 3 Hubei Provincial Key Laboratory of Occurrence and Intervention of Rheumatic Diseases, Hubei Minzu University, Enshi 445000, China; 18871781920@163.com; 4 Hubei Provincial Clinical Medical Research Center for Nephropathy, Minda Hospital of Hubei Minzu University, Enshi 445000, China; 5 School of Public Health, Wuhan University, Wuhan 430071, China

**Keywords:** dihydromyricetin, selenium modification, structure identification, biological activity

## Abstract

Dihydromyricetin, the predominant bioactive constituent in vine tea, manifests diverse bioactivities, including anti-tumoral and anti-inflammatory effects. However, the deep processing of vine tea remains underdeveloped, thereby curtailing its economic benefits. Concurrently, as the demand for organic selenium products escalates, the exploration and development of selenium-containing compounds bearing synergistic effects has emerged as a research frontier. In this investigation, dihydromyricetin underwent selenium modification through a SeO_2_- and HCl-catalyzed reaction, leading to the successful synthesis of selenium-modified dihydromyricetin. A comprehensive array of characterization techniques—encompassing Fourier-transform infrared spectroscopy and solid-state nuclear magnetic resonance—was employed for structural elucidation. The results demonstrated that selenium was covalently tethered to the 4’-hydroxyl group of the B-ring of dihydromyricetin via an O-Se-O bond. Activity assays revealed that selenium-modified dihydromyricetin exhibited significantly augmented inhibitory effects on α-amylase and α-glucosidase (*p* < 0.05) relative to dihydromyricetin, with IC_50_ values of 0.0459 mg/mL and 0.01728 mg/mL, respectively. Moreover, selenium-modified dihydromyricetin exerted marked inhibitory effects on the proliferation of HepG2 and A549 cells, with IC_50_ values of 49.05 μg/mL and 515.60 μg/mL, respectively. These findings collectively furnish experimental evidence underpinning the potential application of selenium-modified dihydromyricetin as a functional food ingredient, particularly within blood glucose regulation.

## 1. Introduction

Dihydromyricetin (DHM), extracted from the traditional medicinal and edible plant vine tea (*Ampelopsis grossedentata*), is a dihydro flavonol with a wide range of pharmacological activities, including anti-inflammatory, antioxidant, anticancer, and immune-enhancing properties. Studies have shown that DHM can effectively alleviate symptoms associated with hypertension, hyperlipidemia, and abnormal blood glucose levels [1]. DHM exhibits remarkable inhibitory effects on the activities of α-amylase (α-AMY) and α-glucosidase (α-GLU), which aids in reducing the hydrolysis of carbohydrates in the diet, thereby lowering postprandial blood glucose levels [2]. Li et al. [3] reported that the IC50 value of DHM for α-GLU was 78.30 μg/mL, and for α-AMY, it was 1.501 mg/mL. Molecular docking analysis indicated that DHM forms multiple hydrogen bonds with key amino acid residues at the active sites of α-GLU and α-AMY, thereby inhibiting their activities. DHM exerts hypoglycemic effects through multi-target regulation of the AMPK/AKT/GSK-3β pathway, which includes enhancing insulin sensitivity, inhibiting hepatic glucose production, and regulating lipid metabolism [4]. Furthermore, various diabetic models have confirmed its systemic effects. The study by Z. Wang et al. [5] demonstrated that DHM could improve sugar–lipid metabolism in diabetic mice by activating the AMPK signaling pathway, reducing blood glucose levels, and decreasing the occurrence of diabetic complications. This suggests that DHM has the potential to be a natural inhibitor for the prevention of postprandial hyperglycemia.

However, the bioactivity of natural DHM still holds significant potential for enhancement. Research has shown that DHM can produce substantial synergistic effects when combined with other drugs, with minimal toxicity or adverse reactions to normal cells and tissues [6]. In recent years, chemical modification has been identified as a pivotal strategy to enhance the bioactivity of DHM-like compounds. Modifications such as glycosylation, acylation, and metal chelation have been shown to improve the bioavailability, solubility, and metabolic stability of DHM [7]. For instance, Garcia et al. [8] employed an enzymatic catalysis method to glycosylate DHM and found that glycosylation could significantly increase its water solubility. In addition, the acylated derivatives they synthesized increased the lipophilicity of DHM, potentially promoting its permeability and bioavailability in a lipid environment. Furthermore, Yao et al. [9] formed DHM–metal (II) complexes with various transition metal ions in air-saturated alkaline solutions and discovered that these complexes exhibited higher antioxidant activity than DHM itself. Interestingly, a small number of researchers have recently focused on the potential synergistic effect of selenium and DHM. For example, Li et al. [10] synthesized selenium nanoparticles modified with DHM, which enhanced their solubility and free radical scavenging capacity, as well as increased the activity of antioxidant enzymes. This modification effectively inhibited apoptosis induced by oxidative stress, showing potential for the treatment of Alzheimer’s disease.

Selenium (Se) is one of the essential trace elements in biological systems, capable of enhancing the activity of superoxide dismutase (SOD) and protecting cells and cell membranes from oxidative damage [11]. SeO_2_ is an inexpensive and readily available source of selenium. It is odorless and tolerant to a wide range of functional groups [12]. SeO_2_ and SeO_3_^2−^ possess roles as pro-oxidants and antioxidants, minimizing oxidative damage by coordinating with metal ions that produce reactive oxygen species (ROS) [13]. Currently, the organic combination of selenium with polysaccharides is an important part of research to achieve the organic modification of inorganic selenium. For instance, Peng et al. [14] synthesized selenium-modified *Codonopsis pilosula* polysaccharides (Se-CPPS) using an optimized microwave-assisted method, and Se is bound to the polysaccharide chain in the following forms of O-Se-O and O-H⋅⋅Se, with valences of 0 or +4. The researchers found that selenium modification enhances the antioxidant activity of *Codonopsis pilosula* polysaccharides and effectively reduces the toxicity of Na_2_SeO_3_. However, research on selenium modification of DHM is limited, and there is a lack of naturally occurring selenium-modified dihydromyricetin (Se-DHM). Consequently, chemical synthesis methods emerge as a pivotal strategy to enhance the yield of Se-DHM.

DHM is the predominant bioactive compound in vine tea and has attracted attention for its diverse pharmacological activities. To maximize the use of DHM and prevent it from being wasted due to tea marketing problems, we explored the chemical modification of DHM. In this study, we pioneered the selenium modification of DHM using the catalytic reaction of SeO_2_ and HCl. The objective of this study was to investigate the structural features and changes in the bioactivity of Se-DHM. Our approach was thoroughly considered. On the one hand, selenium is a trace element with unique bioactivities, which is expected to synergistically enhance the bioavailability and pharmacological value of DHM when combined with DHM, a natural compound with a broad-spectrum pharmacological effect; on the other hand, SeO_2_ and HCl-catalyzed reactions provide relatively mild conditions, avoiding extreme temperatures and pressures. This study offers some insights into optimizing the deep processing of DHM resources and the development of related products. It is hoped that this study will promote the integration of selenium with the vine tea industry to a certain extent, providing new insights into the research and application of selenium-containing bioactive compounds.

## 2. Materials and Methods

### 2.1. Materials and Reagents

DHM (98% purity) was extracted from vine tea and obtained from Shaanxi Xinpai Biotechnology Co., Ltd. (Shaanxi, China), with its purity confirmed by high-performance liquid chromatography (HPLC). GF254 thin-layer chromatography (TLC) silica gel plates were sourced from Qingdao Marine Chemical Factory (Shandong, China). α-AMY and liquid α-GLU were purchased from Shanghai Yuanye Biotechnology Co., Ltd. (Shanghai, China). SeO_2_ (Shanghai Macklin Biochemical Technology Co., Ltd., Shanghai, China). A549 and HepG2 cells were obtained from the Hubei Provincial Key Laboratory of Occurrence and Intervention of Rheumatic Diseases, Hubei Minzu University (Hubei, China). All other reagents used in the experiments were of analytical grade unless otherwise specified.

### 2.2. Preparation of Se-DHM

**Figure 1:**
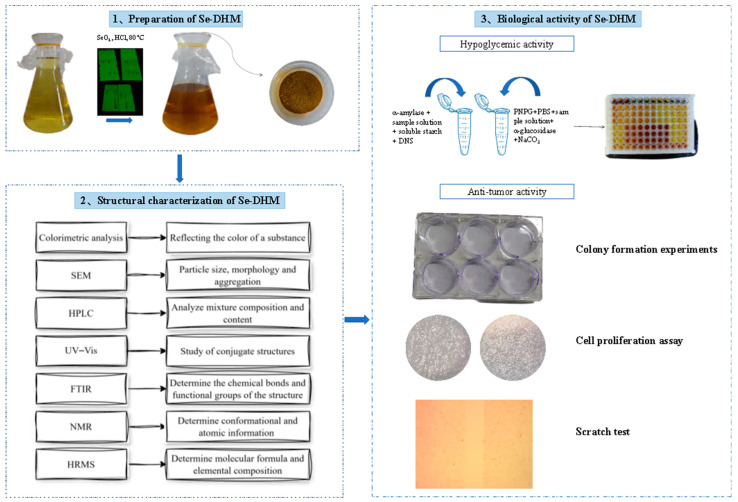
Experimental workflow of Se-DHM preparation and bioactivity assessment.

The Se-DHM was prepared using the method described by Tao et al. [15], with slight modifications. Firstly, introduce 1 g of DHM powder into a clean conical flask, and then add 100 mL of anhydrous ethanol as the solvent. Subsequently, based on the molar ratio of DHM to SeO_2_ (2:1), accurately weigh SeO_2_ and dissolve it in the aforementioned solution. Subsequently, a 5% HCl solution was employed to adjust the pH to 2, and the reaction system was subjected to continuous stirring at 80 °C. The progress of the reaction was monitored using TLC every 30 min, with a mobile phase consisting of hexane–ethyl acetate–acetic acid in a ratio of 10:10:1 (*v*/*v*/*v*), until the characteristic spots of DHM disappeared entirely from the TLC plate, indicating the complete consumption of DHM and the termination of the selenium-modified reaction. After the reaction finished, the system was repeatedly washed with anhydrous ethanol until the washings were colorless and transparent. The filtrate was collected and concentrated. Subsequently, it was washed with ultrapure water until the selenium content in the wash solution was undetectable. The product was then dried to yield the final yellow product, Se-DHM. The selenium content was ascertained through the implementation of atomic fluorescence spectroscopy (AFS-9760, Beijing Haiguang Instrument Co., Ltd., Beijing, China). The overall workflow of this study is outlined in Figure 1.

### 2.3. Structural Identification of Se-DHM

#### 2.3.1. Color Analysis

The color characteristics of DHM and Se-DHM were analyzed using a desktop spectropolarimeter (CS-820N, Hangzhou Color Spectrum Technology Co., Ltd., Hangzhou, China). The L*, a*, and b* values were recorded to quantify the color differences between the two compounds [16].

#### 2.3.2. Morphological Analysis

The morphological features of the dried DHM and Se-DHM samples were examined using a scanning electron microscope (SEM, Apreo 2C, Thermo Fisher Scientific, Waltham, MA, USA) after gold powder fixation at magnifications of ×5000 and ×1000 [17].

#### 2.3.3. Spectroscopic Analysis

Equal amounts of DHM and Se-DHM were dispersed in methanol, and UV–vis (ultraviolet–visible) absorption spectra were obtained using a UV–visible spectrophotometer (CARY 300 Conc, Varian, Palo Alto, CA, USA) in the range of 200–400 nm [18]. The infrared spectra of the samples within the range of 400 to 4000 cm^−1^ were recorded using the potassium bromide pellet method and Fourier-transform infrared spectroscopy (FTIR, Nicolet iS5, Thermo Fisher Scientific, Waltham, MA, USA). Using an X-ray diffractometer (XRD, XRD-7000, Shimadzu Corporation, Kyoto, Japan) at a scanning speed of 8°/min over the range of 10°–80° (2Ɵ) [17].

#### 2.3.4. HPLC of Se-DHM

DHM and Se-DHM powders, along with DHM standards, were further analyzed using HPLC (UltiMate3000, Thermo Fisher Scientific, Waltham, MA, USA) with methanol–0.1% phosphoric acid solution (60:40) as the solvent. The chromatographic column used was a Diamondsil C18 column (4.6 mm × 250 mm, 5 μm, Beijing Dikma Technology Co., Ltd., Beijing, China), with a mobile phase consisting of solvent A: methanol and solvent B: 0.1% phosphoric acid solution, in a ratio of A:B = 32:68 (*v*/*v*), at a flow rate of 0.6 mL/min [19].

#### 2.3.5. Nuclear Magnetic Resonance (NMR) and High-Resolution Mass Spectrometry (HRMS) of Se-DHM

DHM and Se-DHM were dissolved in a DMSO-d6 solution for NMR spectroscopy (Avance III HD, Bruker, Fällanden, Switzerland), while solid-state nuclear magnetic resonance (SSNMR) [20] spectroscopy of Se-DHM was performed (Avance Neo 400WB, Bruker, Berlin, Germany). HRMS were obtained on an ion trap-time of flight mass spectrometer (LCMS-IT-TOF, Shimadzu, Kyoto, Japan) under conditions of an ion accumulation time of 10 ms and a detector voltage of 1.6 kV, calibrated with trifluoroacetic acid and a sodium hydroxide solution, with a scan range of 200–1000 *m*/*z* [21].

### 2.4. In Vitro Hypoglycemic Activity Assay

A total of 0.10 g of Se-DHM and DHM, respectively, was weighed and sample solutions were prepared at various mass concentrations using phosphate buffer solution (PBS, PH = 6.80). The inhibition of α-AMY and α-GLU activities was assessed with modifications to the method described by Qurtam et al. [22], as detailed below:

α-AMY inhibition: Mix 50 μL of α-AMY (2 U/mL) with sample solutions at various concentrations and incubate at 37 °C for 10 min. Add 1 mL of 0.75% soluble starch and incubate for 5 min. Then, add 1 mL of DNS reagent and boil for 5 min. Cool and dilute the solution before measuring absorbance at 520 nm.

α-GLU inhibition: To minimize interference from PNPG oxidation, include a blank control without enzyme. Mix 100 μL of PNPG (10 mmol/L) with 850 μL of PBS and sample solutions, and incubate at 37 °C for 10 min. Add 50 μL of α-GLU (2 U/mL) and incubate for 50 min. Terminate the reaction with 1 mL of Na_2_CO_3_ and measure absorbance at 405 nm.

The inhibition rate is calculated using the following formula (Equation (1)) [23]:

(1) $$\mathrm{Inhibition}\;\mathrm{rate}\;\left(\%\right)=[\left(A-B\right)-\left(C-D\right)]/\left(A-B\right)\times 100$$

where *A* is the absorbance with enzyme but no sample; *B* is the absorbance with neither sample nor enzyme; *C* is the absorbance with both sample and enzyme; and *D* is the absorbance with sample but no enzyme.

### 2.5. Anti-Tumor Activity Experiments

#### 2.5.1. Colony Formation Assay

With slight modifications to the method reported by Jiang et al. [24]. A549 and HepG2 cells were cultured in dishes overnight and then seeded into 6-well plates at 400 cells per well. They were incubated for 48 h in a medium containing various concentrations of DHM and Se-DHM. Cells were then stained with crystal violet for 20 min, and images were captured using an inverted microscope (Axio Vert.A1, Carl Zeiss AG, Oberkochen, Germany). Colony counts were analyzed using ImageJ 1.52a software (National Institutes of Health, Bethesda, MA, USA).

#### 2.5.2. Cell Viability Assay

With minor modifications to the protocol described by Jiang et al. [24], A549 and HepG2 cells (5 × 10^5^ cells/mL) were seeded in 96-well plates and incubated overnight. Test samples were added, and after 24 h, 10 μL of CCK-8 solution was added to each well. After 1 h, absorbance was measured at 450 nm using a microplate reader. The cell survival rate was calculated as Equation (2), outlined below [25]:

(2) $$\mathrm{Cell}\;\mathrm{survival}\;\mathrm{rate}\;\left(\%\right)=[(A1-A2)/(A3-A4)]\;\times \;100$$

where *A*1 is the absorbance with cells and the sample; *A*2 is the absorbance with the sample but without cells; *A*3 is the absorbance with cells but without the sample; and *A*4 is the absorbance without the sample and without cells.

#### 2.5.3. Scratch Assay

The migratory capacity of cells was assessed using a wound-healing assay, as described by Jiang et al. [24] with minor modifications. A549 and HepG2 cancer cell lines, exhibiting good growth, were seeded into 6-well plates at a density of 3 × 10^5^ cells per well and cultured until they reached approximately 90%. A scratch was made in each well, and samples were added. Images were captured at 0, 12, 24, 36, and 48 h using an inverted phase-contrast microscope. The scratch healing rate was calculated as Equation (3), as follows [26]:

(3) $$\mathrm{Scratch}\;\mathrm{healing}\;\mathrm{rate}\;\left(\%\right)=(S0-St/S0)\;\times \;100$$

where *S*0 represents the scratch area at 0 h; *St* represents the scratch area at each time point.

### 2.6. Statistical Analysis

All experiments, unless otherwise specified, were conducted in triplicate. Statistical analyses were performed using GraphPad Prism 9.5.0. All data were analyzed by two-way ANOVA, with Tukey’s test employed to identify significant differences (*p* < 0.05).

## 3. Results

### 3.1. Chroma Analysis

The thermal treatment of DHM solutions can lead to browning due to degradation, oxidation, and polymerization reactions. In this experiment, the amount of DHM involved in browning reactions at 80 °C was relatively limited, allowing a higher proportion of DHM to participate in the formation of adducts [27]. As shown in Table 1, Se-DHM exhibits a reddish-yellow hue, with its brightness (as indicated by the L value) decreasing as the molar ratio of SeO_2_ increases. All products display a reddish-yellow color (both a* and b* values are greater than 0), and significant color differences (Δ*E*) were observed. This phenomenon is likely attributed to the increase in selenium content, which is accompanied by a concomitant change in color [28]. After multiple washes of the reaction system, the selenium content in Se-DHM was determined. The detection of selenium in Se-DHM preliminarily confirms the successful selenium modification of DHM, providing a basis for further characterization.

### 3.2. SEM Analysis

**Figure 2:**
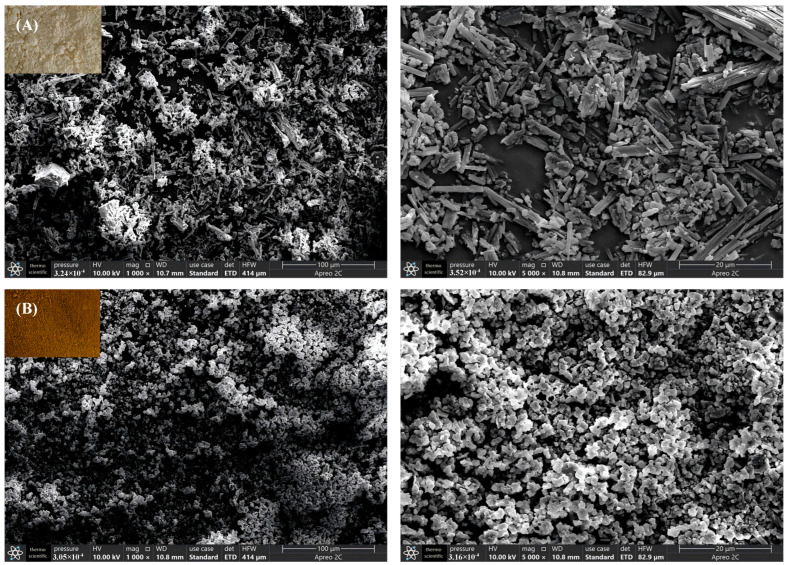
Morphology and microstructure of Se-DHM and DHM: (**A**) photo image of DHM, SEM images of DHM at ×1000 and ×5000 magnification; (**B**) photo image of Se-DHM, SEM images of Se-DHM at ×1000 and ×5000 magnification.

As shown in Figure 2A, DHM exhibits a prismatic crystal morphology under SEM observation at a magnification of ×1000 and ×5000. This characteristic elongated, rod-like shape is attributed to intermolecular interactions between hydroxyl groups in DHM, which facilitate the formation of hydrogen bonding, thereby promoting the development of cylindrical structures. The presence of 2,3-single bonds during the crystallization phase confers significant flexibility, allowing for the formation of imperfect crystalline regions. Consequently, DHM demonstrates high crystallinity and the ability to stack and construct larger organizational structures [29].

In contrast, Figure 2B demonstrates that Se-DHM consists of numerous closely packed small particles that form irregular three-dimensional clusters. This shift in morphology may be due to the fact that the selenide modification reaction occurs on the hydroxyl group of DHM [18], and the selenide modification may disrupt the hydrogen-bonding network, leading to the aggregation of smaller particles and the formation of disordered three-dimensional structures. This change in morphology suggests that selenide modification significantly alters the physical properties of DHM, which may affect its bioavailability and functional properties.

### 3.3. UV−Vis Analysis

**Figure 3:**
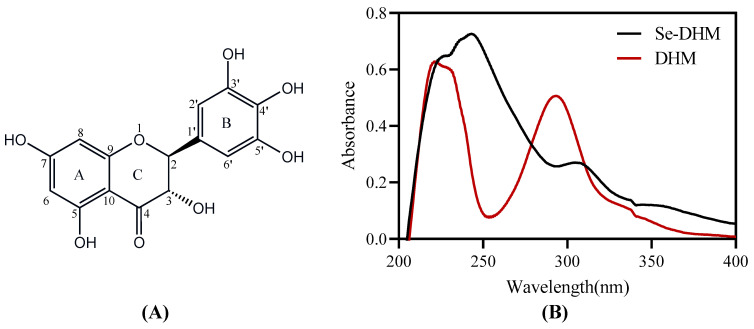
Structural representation and ultraviolet absorption spectra: (**A**) comparative UV−vis absorption spectra of DHM and Se-DHM; (**B**) chemical structure of DHM.

DHM contains a conjugated system composed of a cinnamoyl chromophore (Band I) on the B-ring and a benzoyl group (Band II) on the A-ring, which are typical features of flavonoids and flavonol aglycones. These chromophores manifest in the UV–vis spectrum as absorption bands at 240–285 nm (Band II) and 300–550 nm (Band I) [18]. As shown in Figure 3B, DHM exhibits maximum absorption peaks at 220 nm and 292 nm, while Se-DHM displays maxima at 242 nm and 307 nm. The absorption peak at 220 nm in DHM is attributed to the π-π* transition of the aromatic ring. In contrast, Se-DHM exhibits a strong absorption peak at 242 nm with significantly enhanced absorption, indicating an increased number of conjugated rings, a higher degree of π-conjugation, and the formation of a delocalized conjugated system. The reduction in electron cloud density and the increased electron delocalization in DHM lower the energy required for π→π* transitions, resulting in a redshift of the absorption spectrum [18]. The 15 nm redshift in the UV–vis absorption peak of Se-DHM confirms the formation of a selenium–DHM complex, indicating the structural modification of DHM and implying that the coordination may occur at the cinnamoyl group on the B-ring. Furthermore, selenium modification significantly alters the chromophoric groups and molecular nuclei responsible for coloration, which aligns with the colorimetric results.

### 3.4. HPLC Analysis

**Figure 4:**
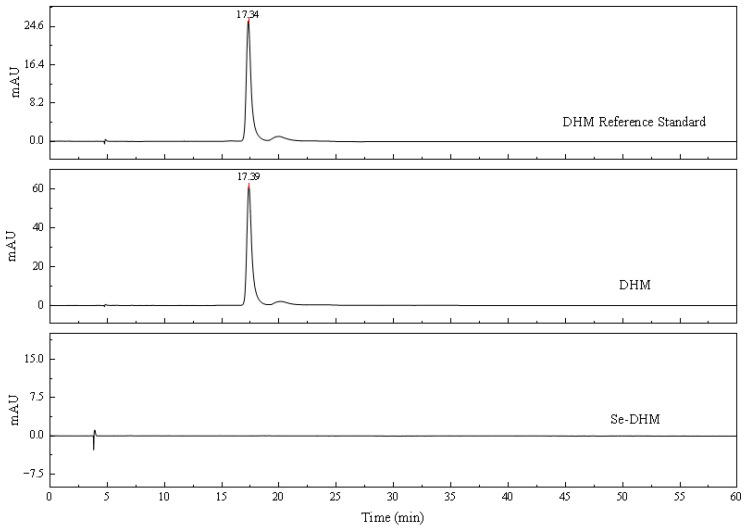
HPLC spectra of DHM and Se-DHM at 240 nm.

During monitoring of the reaction system, TLC indicated the complete disappearance of DHM, suggesting its full conversion. However, multiple product spots were observed. After ethanol washing and rotary evaporation, only a single spot remained at the origin. This suggests that the disappearing substances may be unstable intermediates or compounds that generate polar products. To further analyze this single spot, a C18 solid-phase extraction column was used to collect and analyze Se-DHM by increasing the polarity of the eluent. Although Se-DHM is soluble in methanol, it consistently appeared as a single spot at the origin in both the methanol–chloroform and methanol–water systems. To further investigate, Se-DHM was analyzed by HPLC, as shown in Figure 4. In the HPLC chromatogram of Se-DHM, the characteristic peak of DHM (at 240 nm with a retention time of 17.38 ± 0.01 min) disappeared, and no significant new peaks were observed. Adjustments to the flow rate, column temperature, and mobile phase composition did not result in new peaks. This is likely attributed to the larger atomic radius of selenium and the lack of p-orbital overlap in the Se=O bond, which results in its strong polarity [30]. Additionally, it is possible that the solubility of Se-DHM is still insufficient, which may affect its elution behavior and peak detection in the HPLC system.

Reverse-phase C18 columns are primarily designed for separation based on hydrophobic interactions, making them suitable for nonpolar or moderately polar compounds [31]. However, for compounds with polar functional groups, adsorption mechanisms dominate the retention process. The separation efficiency of Se-DHM is influenced by its polarity and solubility on the stationary phase surface. In cases where the sample is polar or strongly polar, interactions with the nonpolar bonding phase of the C18 column may be insufficient, resulting in short retention times, failure to elute, or peaks near the dead time, thereby affecting separation efficiency [32]. Ali et al. [33] reported poor separation of curcumin on C18 columns, characterized by poorly resolved and non-sharp peaks, which resulted in unreported or excessively high detection limits. Similarly, Accucore columns, despite offering relatively quick separation, are unable to effectively separate curcumin under typical laboratory conditions. Lu et al. [34] noted that in their experiments, no peaks were observed when using acetonitrile–water as the mobile phase due to its weak elution strength, which led to the retention of carbon quantum dots (CQDs) on the column. When employing methanol and water as the mobile phase, the signal of the peaks was weak, with many CQDs still retained on the C18 column.

### 3.5. FTIR and XRD Analyses

**Figure 5:**
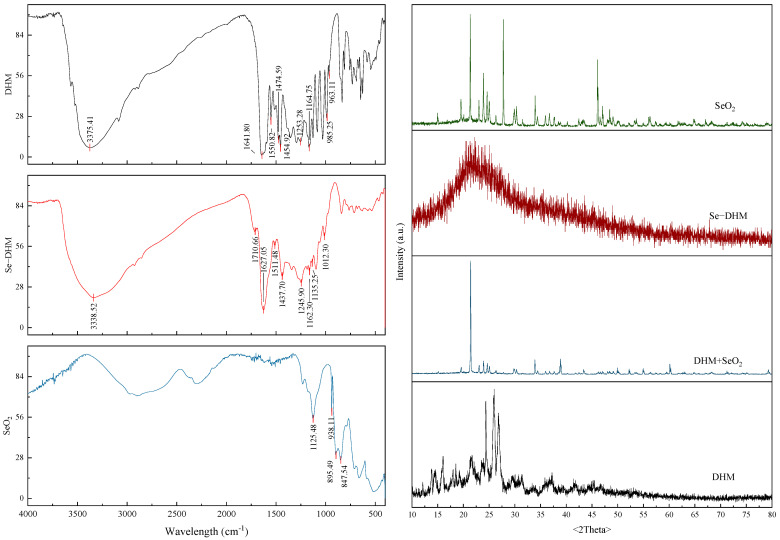
FTIR spectra (**left**) and XRD patterns (**right**) of DHM and Se-DHM.

The FTIR spectra of Se-DHM and DHM are shown in Figure 5. DHM exhibits characteristic peaks at 3375 cm⁻^1^ (O-H stretching vibration), 1641 cm⁻^1^ (C=O stretching vibration), 1551, 1475, and 1455 cm⁻^1^ (aromatic ring stretching vibrations), and 1253 and 1164 cm⁻^1^ (C-O-C stretching vibrations) [35]. In Se-DHM, the peak at 3339 cm⁻^1^ shows reduced intensity and broadening, indicating that free hydroxyl groups are likely the primary interaction sites. The formation of hydrogen bonds between Se-DHM molecules may attenuate their stretching vibrations [36]. Selenium readily interacts with the O, C, and N groups, leading to shifts in some FTIR peaks, particularly in the C-O characteristic region [37]. The new absorption peak of Se-DHM at 1012.30 cm⁻^1^ is attributed to the O-Se-O bond, which is consistent with the reported range of 1010–1040 cm⁻^1^ [14].

The crystal structure of Se-DHM was characterized using XRD, as shown in Figure 5. The absence of sharp diffraction peaks indicates a low degree of crystallinity, suggesting that Se-DHM exhibits amorphous or disordered structures. These findings imply that Se-DHM predominantly exists in polycrystalline and amorphous forms, with a more disordered chain arrangement. This structural disorder may result from the formation of new selenium-containing polymers with varying degrees of compactness, creating a denser network structure. The formation of hydrogen bonds in Se-DHM likely contributes to its lower crystallinity compared to DHM [35]. The DHM + SeO_2_ mixture exhibits distinct crystal diffraction peaks of both components, unlike Se-DHM, indicating that it is formed via a chemical reaction rather than a simple physical mixture.

### 3.6. HRMS and NMR Analysis

**Figure 6:**
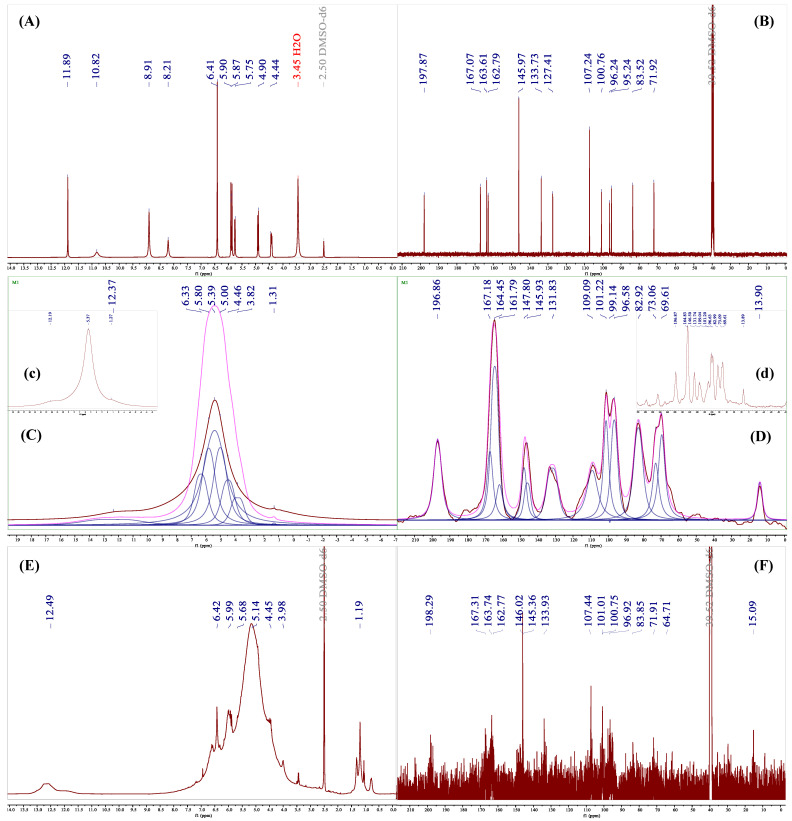
NMR and SSNMR spectra of DHM and Se-DHM: (**A**,**B**) represent the 1H and 13C NMR spectra of DHM, respectively; (**C**,**c**,**D**,**d**) represent the fitted H and C SSNMR spectra of Se-DHM; (**E**,**F**) represent the 1H and 13C NMR spectra of Se-DHM.

**Figure 7:**
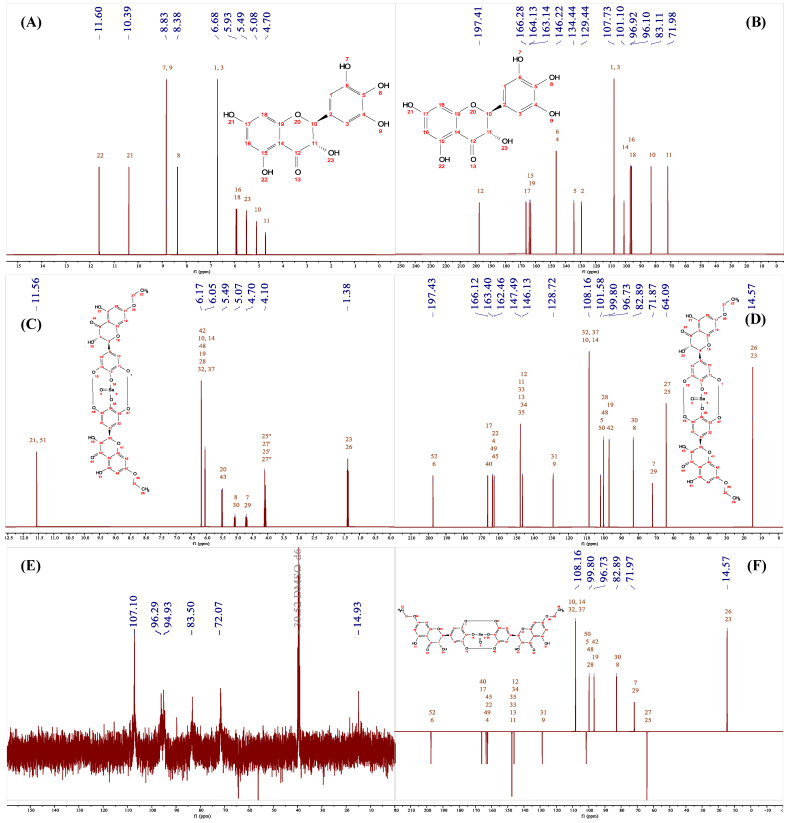
Predicted spectra of DHM and Se-DHM and the DEPT135 spectrum of Se-DHM: (**A**,**B**) represent the predicted H and C spectra of DHM; (**C**,**D**) show the verification spectra of the H and C NMR spectra of Se-DHM; (**E**,**F**) present the DEPT135 spectrum and its verification spectrum of Se-DHM.

The NMR spectra of DHM are presented in Figure 6A,B, with the following chemical shift characteristics: In 1H NMR (400 MHz, DMSO-d6), δ 11.89 (s, 5H, OH), 10.82 (s, 7H, OH), 8.91 (s, 3′H+5′H, OH), 8.21 (s, 4′H, OH), 6.41 (s, 2′H+6′H, OH), 5.90 (d, 8H, H), 5.87 (d, 6H, H), 5.75 (d, 3H, OH), 4.90 (d, 2H, H), 4.44 (dd, 3H, H). These findings are consistent with previous reports [18]. The 13C NMR (400 MHz, DMSO-d6) reveals peaks at δ 197.87 (C-4), 167.07 (C-7), 163.61 (C-5), 162.79 (C-9), 145.97 (C-3′,5′), 133.73 (C-4′), 127.41 (C-1′), 107.24 (C-2′,6′), 100.76 (C-10), 96.24 (C-6), 95.24 (C-8), 83.52 (C-2), and 71.92 (C-3). The high degree of consistency between the observed and predicted spectra in Figure 6A,B, particularly the C spectrum in Figure 7B, indicates minimal prediction error and confirms their applicability for subsequent structural elucidation.

However, Se-DHM exhibits solubility issues in common deuterated solvents, as shown in Figure 6E,F. To address this, an SSNMR analysis was conducted. The C spectra of Se-DHM and DHM, depicted in Figure 6F, show negligible differences, suggesting that the carbon framework of Se-DHM remains largely unchanged. Conversely, the H spectrum of Se-DHM in Figure 6C,D shows the disappearance of peaks after δ 6.41, indicating the involvement of hydroxyl groups on rings A and B in the reaction. Comparison of the H and C spectra of Se-DHM with those of DHM reveals new peaks at δ 1.31 in the H spectrum and δ 13.90 in the C spectrum. The positive peak at this position in Figure 7E suggests the presence of a methyl group [38]. Additionally, the peak at δ 69.61 in the SSNMR of Se-DHM corresponds to δ 64.71 in the NMR, identified as a negative peak in the DEPT135 spectrum, indicating a -CH_2_- structure. Based on Figure 7C,D, it is inferred that a -CH_2_-CH_3_ substitution occurred at the C7-OH position, and hydrogen bonds may have formed at the 3′-OH and 5′-OH positions [39]. Spectral analysis predicts that if selenation occurred at the 7-C position, the peak at δ 96.63 (6-C) would disappear, leading to the conclusion that selenation modification took place at the hydroxyl group on the 4′-C of ring B. The proton absorption peak of C5-OH in DHM is located at δ 11.89, which shifted to δ 12.19 upon complex formation. This shift is analogous to the reported shift of the C5-OH peak in luteolin from 12.94 to 13.36 upon lead complexation, likely due to electron transfer from near the C5-OH to the selenium atom, resulting in reduced electron density [40]. The upfield shift of the complex’s chemical shift compared to DHM is attributed to the conjugation effect during selenium complexation [41], indicating that C5-OH did not participate in the reaction.

**Figure 8:**
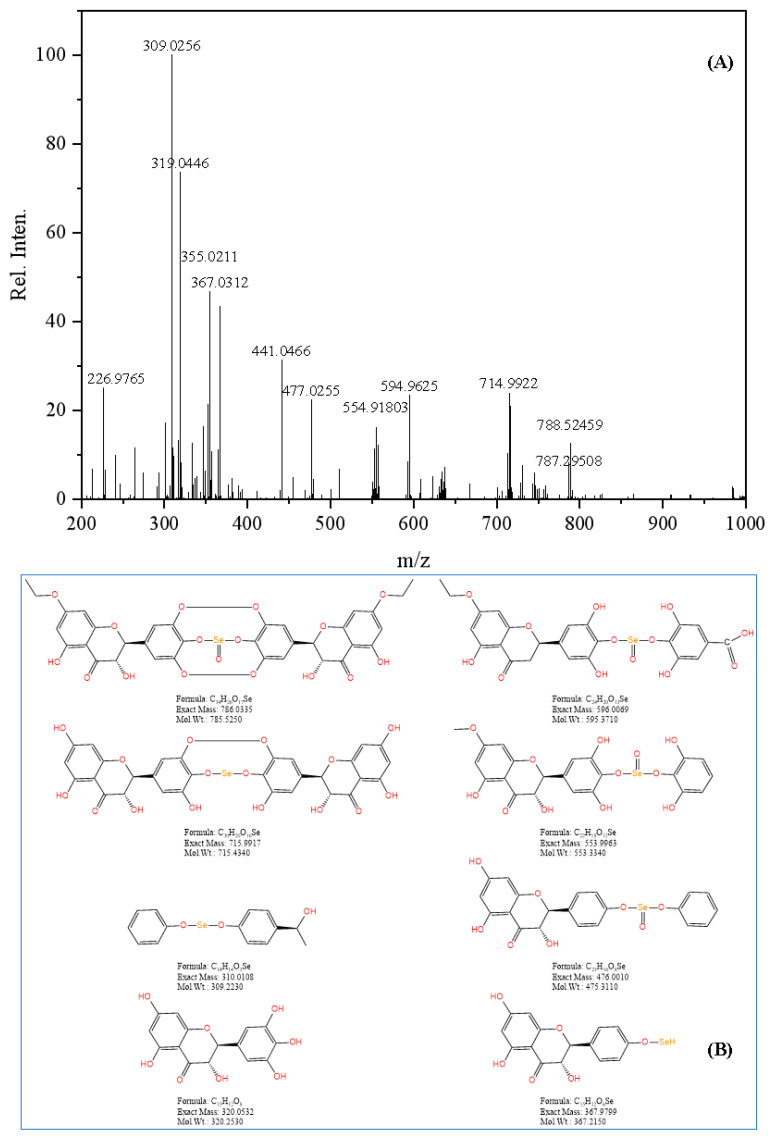
HRMS spectrum of Se-DHM: (**A**) HRMS spectrum of Se-DHM; (**B**) mass spectrometry fragmentation structure of Se-DHM.

As shown in Figure 8A, HRMS analysis in the range of 100 to 1000 *m*/*z* showed a peak at *m*/*z* = 787.29508, corresponding to the elemental composition of C34H26O17Se. The proposed structure was further supported by mass spectrometry fragmentation analysis in Figure 8B. These findings indicate that the mass spectrum of Se-DHM in the range of 100 to 1000 *m*/*z* consists of a 2:1 ratio of DHM to Se, consistent with the preceding NMR analysis. It should be noted that due to the limitations of the mass spectrometry analysis, only local structural information of the molecule is provided, and it does not represent the overall molecular weight of Se-DHM.

### 3.7. Blood-Glucose-Lowering Ability

The differential inhibitory effects of Se-DHM and natural DHM on α-AMY and α-GLU underscore the critical role of structural modification in enhancing enzyme inhibition. α-AMY—primarily secreted by the salivary glands and pancreas—catalyzes the hydrolysis of starch into oligosaccharides, which are subsequently further cleaved into absorbable monosaccharides by α-GLU in the intestinal mucosa. Inhibiting these enzymes represents a strategic approach to mitigating postprandial blood glucose peaks, a key objective in diabetes management [22].

**Figure 9:**
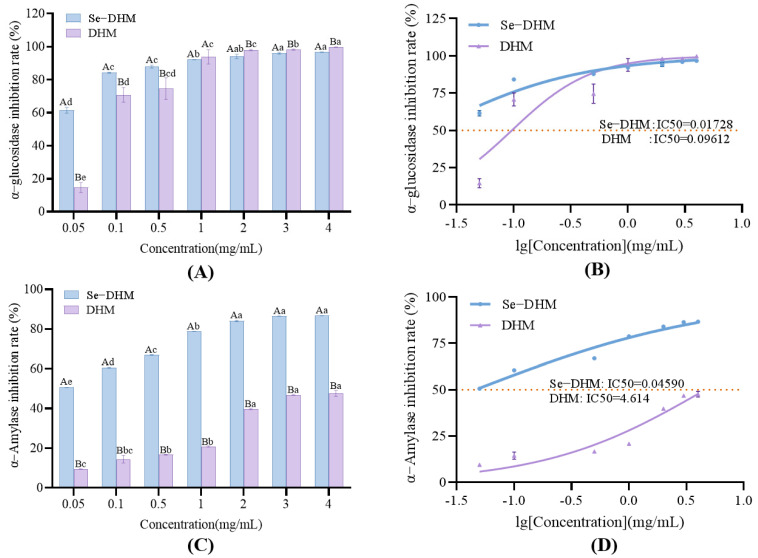
The ability of Se-DHM and DHM to inhibit α-AMY and α-GLU: (**A**) shows the inhibition rate of Se-DHM and DHM on α-GLU; (**B**) presents the corresponding IC50 values; (**C**) presents the inhibition rate of Se-DHM and DHM on α-AMY; (**D**) shows the respective IC50 values. In the figures, lowercase letters indicate significant differences within groups, while uppercase letters denote significant differences between groups (*p* < 0.05).

As illustrated in Figure 9, both Se-DHM and DHM demonstrated dose-dependent inhibition of α-AMY within the concentration range of 0.05–4.00 mg/mL, with saturation kinetics observed at concentrations exceeding 2.00 mg/mL. The saturation of inhibition at higher concentrations (>2.00 mg/mL) may reflect limitations in the occupancy of the enzyme active site or a tendency for Se-DHM to aggregate at elevated doses [42]. The IC50 value of Se-DHM (0.0459 mg/mL) was significantly lower than that of DHM (4.6140 mg/mL). A similar trend was observed for α-GLU inhibition, with Se-DHM exhibiting an IC50 of 0.01728 mg/mL, significantly lower than the 0.09612 mg/mL for DHM (approximately a 5.6-fold improvement). This enhancement is likely attributed to the incorporation of selenium, which optimizes molecular interactions with the enzyme active site. The differences in enhancement between the two enzymes suggest that the structure–activity relationship is influenced by the distinct catalytic mechanisms of α-AMY and α-GLU [43]. The pronounced inhibitory activity of Se-DHM may arise from a synergistic effect. The larger atomic radius and higher electron density of the selenium atom facilitate stable interactions with the enzyme’s active site, thereby inhibiting enzyme activity. The catalytic domain of α-AMY, characterized by a rich β-sheet structure, contains aspartic acid residues (Asp197 and Asp300) that are critical for substrate binding. The redox-active surface of selenium may stabilize interactions with these residues, thereby obstructing substrate access, a phenomenon corroborated by molecular docking studies in analogous systems [14].

### 3.8. Anti-Tumor Activity

**Figure 10:**
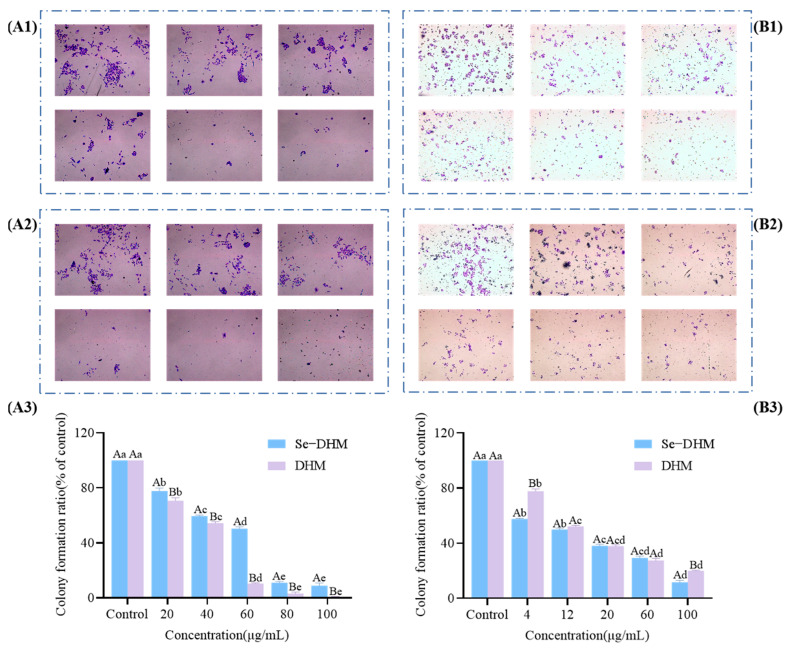
Crystal violet staining results: (**A**) shows the results of A549 cells, with (**A1**–**A3**), respectively, representing the microscopic images of DHM-treated A549 cells, the microscopic images of Se-DHM-treated A549 cells, and the colony formation rate of A549 cells; (**B**) shows the results of HepG2 cells, with (**B1**–**B3**), respectively, representing the microscopic images of DHM-treated HepG2 cells, the microscopic images of Se-DHM-treated HepG2 cells, and the colony formation rate of HepG2 cells. In the figures, lowercase letters indicate significant differences within groups, while uppercase letters denote significant differences between groups (*p* < 0.05).

As illustrated in Figure 10, the results of the crystal violet staining demonstrated that both Se-DHM and DHM exhibited inhibitory effects on the proliferation of HepG2 and A549 cells, with a positive correlation to the dose and duration of treatment. Specifically, as the concentrations of Se-DHM and DHM increased and the exposure time lengthened, the proliferation of HepG2 and A549 cells was increasingly suppressed. These findings suggest that Se-DHM and DHM may inhibit cell proliferation through mechanisms such as cell cycle arrest or apoptosis induction. The findings further underscore the necessity for comprehensive consideration of drug concentration and exposure time in subsequent mechanistic studies to elucidate the molecular mechanisms underlying the anti-proliferative effects of Se-DHM and DHM.

**Figure 11:**
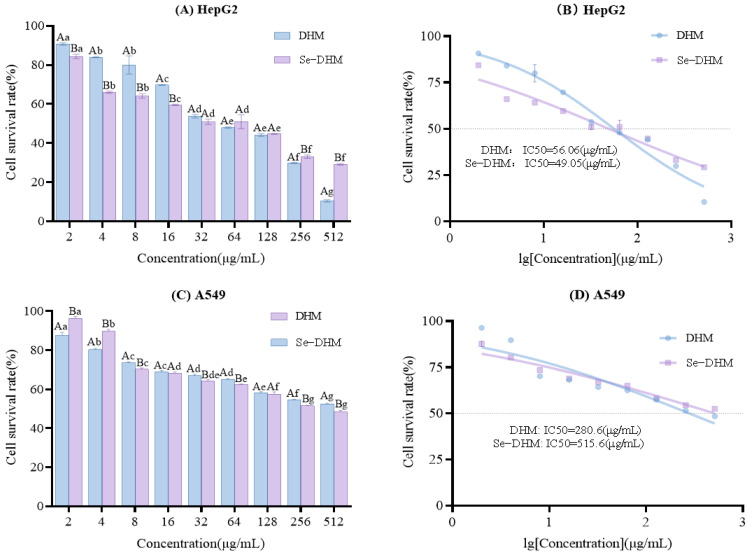
Effects of Se-DHM and DHM on cell survival rate and IC50 values in HepG2 and A549 cells: (**A**) shows the cell survival rate after Se-DHM and DHM treatment in HepG2 cells; (**B**) presents the IC50 values of Se-DHM and DHM in HepG2 cells; (**C**) shows the cell survival rate of A549 cells treated with Se-DHM and DHM; (**D**) displays the IC50 values of Se-DHM and DHM in A549 cells. In the figures, lowercase letters indicate significant differences within groups, while uppercase letters denote significant differences between groups (*p* < 0.05).

To quantitatively assess the impact of Se-DHM on the proliferation of HepG2 and A549 cells, the CCK-8 proliferation assay was conducted, as presented in Figure 11. The assay revealed that both Se-DHM and DHM potently inhibited the in vitro proliferation of HepG2 and A549 cells, with the inhibitory effect escalating in conjunction with increased mass concentrations. Specifically, the IC50 values for DHM against HepG2 and A549 cells were 56.06 μg/mL and 280.60 μg/mL, respectively, whereas the IC50 values for Se-DHM against these cell lines were 49.05 μg/mL and 515.60 μg/mL, respectively. These data suggest that Se-DHM exhibits a marginally greater efficacy in inhibiting HepG2 cell proliferation compared to DHM; however, it is less effective in inhibiting A549 cell proliferation. This disparity may be associated with the alteration of hydroxyl group structures due to the introduction of selenium in Se-DHM. Chidambara Murthy et al. [44] have identified hydroxyl groups at the C3 and C6 positions, ortho-hydroxylation of the B-ring, and a double bond between C2 and C3 as significant structural features for cancer cell inhibition. While ortho-positioned hydroxyl groups are crucial for anti-proliferative activity, the presence of a C5-C6 ortho-hydroxyl group further enhances the potency of flavonoids.

**Figure 12:**
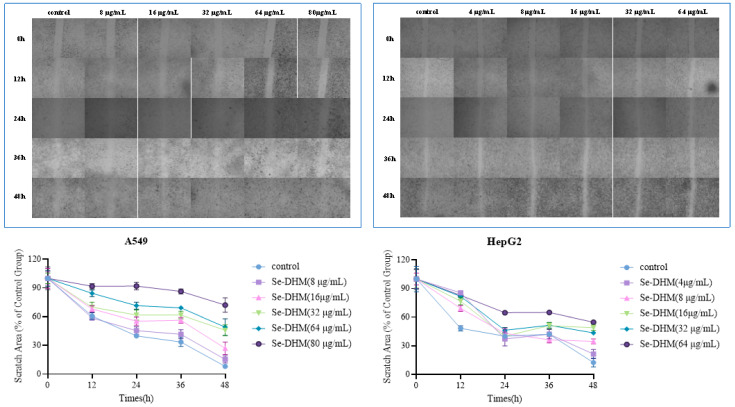
Results of the wound-healing assay of A549 and HepG2 cells treated with Se-DHM.

**Figure 13:**
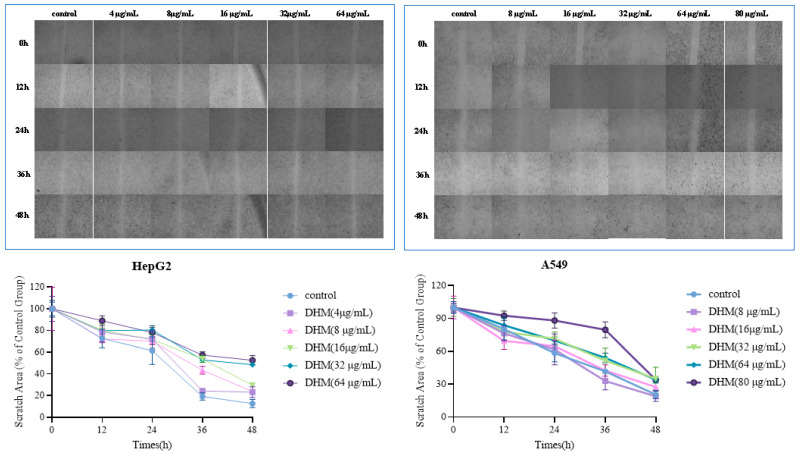
Results of the wound-healing assay of A549 and HepG2 cells treated with DHM.

As illustrated in Figure 12 and Figure 13, the wound-healing assay results demonstrate that the inhibitory effects of Se-DHM on the migration of two cell lines increase with increasing concentration and time. Moreover, these effects are significantly superior to those of DHM. This is mainly because Se-DHM can inhibit the expansion and metastasis of cell populations by inducing apoptosis [45], which further confirms that Se-DHM can enhance the inhibition of the migratory ability of HepG2 and A549 cells and improve anti-tumor activity. These findings provide experimental evidence for the effects of Se-DHM on cancer cell growth and migration. Moreover, these results further verify that Se-DHM can significantly enhance the inhibition of the migration of HepG2 and A549 cells, thereby improving its anti-tumor activity and offering strong experimental support for the application of Se-DHM in anti-tumor treatment.

## 4. Discussion

Recent studies have demonstrated that the combination of selenium and flavonoids can significantly enhance biological activity. For instance, Gutiérrez et al. [46] found that the complex of selenium nanoparticles with luteolin and dioscin could improve insulin sensitivity and glucose utilization, thereby exerting a better hypoglycemic effect in diabetic models than the traditional drug glibenclamide. Similarly, Zaghloul et al. [47] reported that selenium–rutin nanoparticles showed a significant regulatory effect on blood glucose levels in diabetic rats. These studies collectively indicate that the introduction of selenium, through chemical bonding or nano-complexation, can optimize the bioavailability and targeting of flavonoids, thereby amplifying their hypoglycemic activity.

In this study, Se-DHM was synthesized via the reaction of DHM with SeO_2_. Characterization of Se-DHM was performed using FTIR, HRMS, and SSNMR. The results revealed that Se in Se-DHM was connected to the 4′-OH of DHM’s B-ring via an O-Se-O bond. Moreover, Se-DHM exhibited remarkable inhibitory effects on α-AMY and α-GLU, with IC₅₀ values of 0.0459 mg/mL and 0.01728 mg/mL, respectively. These findings suggest that Se-DHM has a significant advantage in regulating blood glucose levels by inhibiting the activity of these two enzymes.

Based on the structural features of Se-DHM and the literature precedents, the type of inhibition may involve either competitive or hybrid inhibition. Flavonoids usually achieve competitive inhibition through the formation of hydrogen bonds and hydrophobic interactions with the active sites of enzymes [48]. The structure of Se-DHM contains hydroxyl and carbonyl groups that are capable of participating in the formation of hydrogen bonds, which provides the possibility of competitive binding to the active sites of enzymes. In addition, the introduction of selenium at the 4′-OH position (via O-Se-O bonds) may enhance this interaction. Selenium, with its large atomic radius and polarizability, may further enhance this competitive inhibition by changing the polarity or charge distribution of the molecule [49]. In addition, Se-DHM may bind to allosteric sites, inducing conformational changes that reduce enzyme activity [50]. For example, methoxy on the A-ring and selenium on the B-ring may interact with the peripheral hydrophobic region, thereby altering the structure of the enzyme [51]. This is consistent with previous studies on selenium-modified flavonoids, which have shown stronger hypoglycemic activity. For example, Deng et al. [52] reported that selenium-loaded luteolin nanoparticles exhibited comprehensive characteristics in blood glucose regulation, enhancing intestinal permeability and trans-epithelial transport, alleviating oxidative stress responses, improving pancreatic function, and increasing glucose utilization in adipose tissue. In addition, Se-DHM showed a more pronounced inhibitory effect on cell proliferation than DHM.

Despite the encouraging results of this study, the hypoglycemic and anti-tumor mechanisms of Se-DHM remain unclear. Further research is needed to explore the molecular mechanisms and in vivo efficacy of Se-DHM. Additionally, the long-term safety and toxicity of Se-DHM require more comprehensive evaluation in future studies.

## Figures and Tables

**Table 1:** Chroma analysis of Se-DHM.

Sample	L*	a*	b*	Δ*E*	Selenium Content (%)
n(DHM:SeO_2_) = 1:3	54.73 ± 0.10 ^d^	10.20 ± 0.39 ^a^	20.42 ± 0.20 ^c^	50.70 ± 0.10 ^a^	15.89 ± 0.20 ^a^
n(DHM:SeO_2_) = 1:2	62.84 ± 0.00 ^c^	8.04 ± 0.01 ^c^	23.05 ± 0.01 ^b^	44.46 ± 0.00 ^b^	12.42 ± 0.12 ^b^
n(DHM:SeO_2_) = 1:1	62.73 ± 0.60 ^c^	8.77 ± 0.04 ^b^	30.09 ± 0.60 ^a^	48.70 ± 0.09 ^c^	7.61 ± 0.13 ^c^
n(DHM:SeO_2_) = 2:1	74.82 ± 0.17 ^b^	7.26 ± 0.01 ^d^	30.16 ± 0.18 ^a^	39.96 ± 0.03 ^d^	5.51 ± 0.07 ^d^
n(DHM:SeO_2_) = 3:1	78.72 ± 0.13 ^a^	3.41 ± 0.02 ^e^	30.36 ± 0.66 ^a^	37.24 ± 0.46 ^e^	4.61 ± 0.11 ^e^
DHM	94.45 ± 0.28	−2.06 ± 0.14	10.25 ± 0.40	11.84 ± 0.43	0

Note: L* denotes lightness and darkness (black and white); a* denotes red–green; and b* denotes yellow–blue. In the table, different lowercase letters indicate significant differences among groups (*p* < 0.05).

## Data Availability

Data are not available in public datasets; please contact the authors.

## Abbreviations

The following abbreviations are used in this manuscript:

α-AMY	α-amylase
α-GLU	α-glucosidase
CQDs	Carbon quantum dots
DHM	Dihydromyricetin
FTIR	Fourier-transform infrared spectroscopy
HPLC	High-performance liquid chromatography
HRMS	High-resolution mass spectrometry
NMR	Nuclear Magnetic Resonance
PBS	Phosphate buffer solution
ROS	Reactive oxygen species
Se	Selenium
Se-CPPS	Selenium-modified Codonopsis pilosula polysaccharides
Se-DHM	Selenium-modified dihydromyricetin
SEM	Scanning electron microscope
SOD	Superoxide dismutase
SSNMR	Solid-state nuclear magnetic resonance
TLC	Thin-layer chromatography
UV−Vis	Ultraviolet−visible
XRD	X-ray diffractometer
